# Modeling the Accuracy of Two *in-vitro* Bovine Tuberculosis Tests Using a Bayesian Approach

**DOI:** 10.3389/fvets.2019.00261

**Published:** 2019-08-13

**Authors:** Catalina Picasso-Risso, Andres Perez, Andres Gil, Alvaro Nunez, Ximena Salaberry, Alejandra Suanes, Julio Alvarez

**Affiliations:** ^1^Department of Veterinary Population Medicine, University of Minnesota, Saint Paul, MN, United States; ^2^Facultad de Veterinaria, Universidad de la Republica, Montevideo, Uruguay; ^3^División Laboratorios Veterinarios “Miguel C. Rubino”, Ministerio de Ganadería, Agricultura y Pesca, Montevideo, Uruguay; ^4^VISAVET Health Surveillance Centre, Universidad Complutense, Madrid, Spain; ^5^Departamento de Sanidad Animal, Facultad de Veterinaria, Universidad Complutense de Madrid, Madrid, Spain

**Keywords:** latent class analysis, diagnosis, interferon-gamma release assay, elisa, chronically infected, Uruguay

## Abstract

Accuracy of new or alternative diagnostic tests is typically estimated in relation to a well-standardized reference test referred to as a gold standard. However, for bovine tuberculosis (bTB), a chronic disease of cattle, affecting animal and public health, no reliable gold standard is available. In this context, latent-class models implemented using a Bayesian approach can help to assess the accuracy of diagnostic tests incorporating previous knowledge on test performance and disease prevalence. In Uruguay, bTB-prevalence has increased in the past decades partially because of the limited accuracy of the diagnostic strategy in place, based on intradermal testing (caudal fold test, CFT, for screening and comparative cervical test, CCT, for confirmation) and slaughter of reactors. Here, we evaluated the performance of two alternative bTB-diagnostic tools, the interferon-gamma assay, IGRA, and the enzyme-linked immunosorbent assay (ELISA), which had never been used in Uruguay in the absence of a gold standard. In order to do so animals from two heavily infected dairy herds and tested with CFT-CCT were also analyzed with the IGRA using two antigens (study 1) and the ELISA (study 2). The accuracy of the IGRA and ELISA was assessed fitting two latent-class models: a two test-one population model (LCA-a) based on the analysis of CFT/CFT-CCT test results and one *in-vitro* test (IGRA/ELISA), and a one test-one population model (LCA-b) using the IGRA or ELISA information in which the prevalence was modeled using information from the skin tests. Posterior estimates for model LCA-a suggested that IGRA was as sensitive (75–78%) as the CFT and more sensitive than the serial use of CFT-CCT. Its specificity (90–96%) was superior to the one for the CFT and equivalent to the use of CFT-CCT. Estimates from LCA-b models consistently yielded lower posterior Se estimates for the IGRA but similar results for its Sp. Estimates for the Se (52% 95%PPI:44.41-71.28) and the Sp (92% 95%PPI:78.63–98.76) of the ELISA were however similar regardless of the model used. These results suggest that the incorporation of IGRA for detection of bTB in highly infected herds could be a useful tool to improve the sensitivity of the bTB-control in Uruguay.

## Introduction

Accuracy of diagnostic tests has been traditionally estimated by comparing the test results with those of a reference test, sometimes referred to as the gold standard, which unequivocally indicates the true status of an individual (infected/not infected). In the absence of such a reference test, latent class analyses based on Bayesian methods provide an alternative strategy for evaluation of diagnostic tests when the true status of the individual is unknown. The use of this approach in the context of veterinary medicine has been described elsewhere (1). Briefly, the use of latent class analyses based on Bayesian methods involves the combination of previous knowledge on test performance (when available) with the evidence provided by newly collected data to obtain a posterior estimate on test performance and disease prevalence, often achieved through Monte Carlo simulations using Gibbs sampling (2). The prior knowledge on test performance is typically obtained through the review of the scientific literature and/or the elicitation of expert opinion (3). Methodologies to elicit expert opinion have been described elsewhere (3).

Use of latent class models in veterinary epidemiology has increased in the past decades, particularly for the assessment of diagnostic tests for chronic and complex diseases for which gold standard tests are not available, such as bovine tuberculosis (bTB) (4–7).

Bovine tuberculosis, mainly caused by infection with *Mycobacterium bovis* (*M. bovis*), is an important chronic disease of cattle that causes a substantial impact on animal and public health, and that imposes a significant economic burden associated to its control and international trade restrictions (8, 9).

Control programs worldwide are based on test and removal of positive animals or, in some cases, complete herds (10). In Uruguay, the bTB-national program involves serial intradermal testing (caudal fold test –CFT- followed by the comparative cervical test –CCT- for confirmation) of all dairy herds annually for the detection of infected animals and its posterior removal (11, 12). In the past decade, the number of bTB-positive dairy herds detected every year, the within-herd prevalence in infected farms, and the time from outbreak detection to control has increased in Uruguay despite measures implemented as part of the national bTB control program (12, 13). The evolution of the dairy industry in the country, characterized by an increase in herd sizes and production intensification, has been associated with the limited success of bTB-control in recent years (12, 14). Additionally, insufficient sensitivity of bTB diagnostic tests may also contribute to the persistence of potentially infectious individuals in the herd that can further spread the disease within and between herds (15).

In Europe the use of the interferon-gamma release assay (IGRA) in parallel with the skin test has been incorporated in many eradication programs to maximize diagnostic sensitivity (Council Directive 64/432/EEC, 1964) (16, 17). Other tests based in the detection of specific antibodies (such as the enzyme-linked immunosorbent assay, ELISA) have been developed and proven useful for detection of specific subpopulations of *M. bovis*-infected animals that may not react to the skin test, although their field use has been mostly limited so far to experimental purposes (18–23). Differences in the performance of bTB diagnostic tests can be related with local factors related with the personnel conducting the tests (experience) or with the cattle population (frequency of testing, presence of other diseases compromising the immune response, breed, among others). Characterization of the performance of alternative diagnostic tools (IGRA and ELISA) previously never implemented in Uruguay may help to design strategies for the improvement of the diagnostic sensitivity in high bTB-prevalence infected dairy herds, currently a priority for the control and eradication of bTB in the country.

Here, we aimed to estimate the accuracy of two commercial assays for *in-vitro* diagnosis of bTB that had never been used in Uruguay, namely an IGRA (using two alternative antigens –referred to as study1-) and an antibody-based ELISA (referred to as study 2), fitting two different latent-class models in a Bayesian framework. Results from this research will help to quantify the potential impact that alternative diagnostic strategies may have in improving the effectiveness of the bTB-control program in Uruguay.

## Methods

We followed the STARD-BLCM guidelines to describe the materials and methods in our study (24).

### Study Design and Source Population

**Figure 1:**
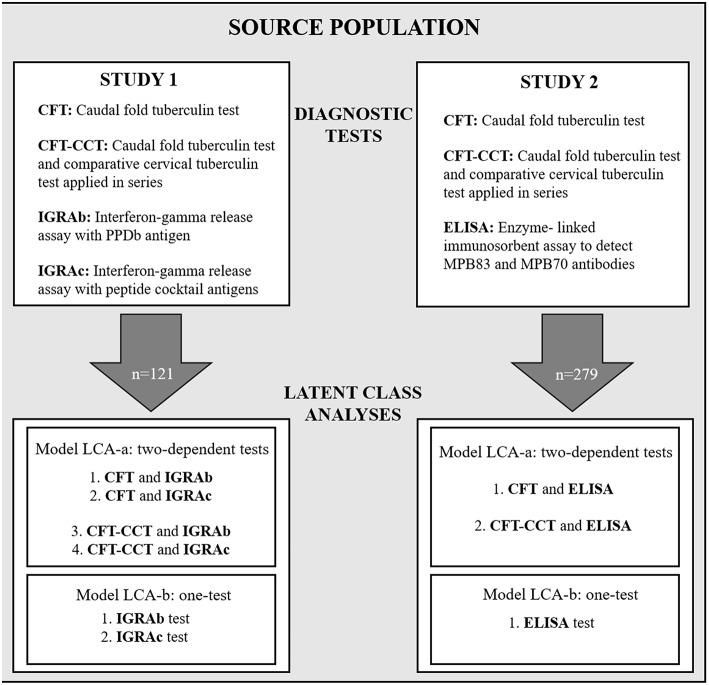
Schematic diagram showing the study design, with the diagnostic tests used for study 1 and study 2, and the Bayesian latent-class fitted models LCA-a and LCA-b.

Two cross-sectional diagnostic accuracy studies (referred to as study 1 and study 2 –Figure 1) using a “single-gate” diagnostic design were performed to evaluate the performance of the *in-vitro* bTB-diagnostic assays (25).

Sampling for both studies was carried in 2016, and included 121 and 279 Holstein cows for studies 1 and 2, respectively. All animals were selected from two commercial dairy herds belonging to the same company (with similar management practices and that frequently and systematically mingle their animals) located in the Department of Florida. Both herds were bTB positive since 2013. The two herds were subjected to the intradermal test as regulated by the national bTB-control program in Uruguay for dairies based on the status of the herd (26). In addition, blood and serum samples were drawn from the selected animals (Figure 1). For logistics reasons blood samples from 158 animals were not collected reducing the sample size for study 1 in comparison to study 2. To avoid selection bias, the proportion of CFT-positive results in animals with missing blood samples in comparison to the ones that have blood samples for study 1 were assessed using a Pearson's chi-square test.

### Sampling and Diagnostic Assays

All dairy >12 month animals were tested using the CFT as a screening test, involving the intradermal inoculation of a purified protein derivate from *M. bovis* (PPDb) in the caudal area. Animals with an increase in skin thickness and/or presence of *in-situ* clinical signs of inflammation 72 h post inoculation were considered reactors and subjected to the CCT for confirmation within the following seven days. In this test, two PPD inoculations from *M. bovis* (PPDb) and *M. avium* (PPDa) are performed in the cervical area. When the difference in skinfold thickness in the PPDb inoculation site was ≥4 mm than the PPDa animals were considered infected and culled.

Blood samples from the coccygeal vein of cows enrolled in studies 1 and 2 were collected in tubes with (IGRA) or without (antibody ELISA) heparin, respectively, after the results of the serial CCT test were assessed (if applicable). Blood samples were maintained in a container at monitored environmental temperatures (22 ± 3°C) until arrival to the official veterinary diagnostic laboratory (Miguel C. Rubino) within the first 8 h post extraction to perform the IGRA (Prionics, Schlieren-Zurich, Switzerland). Serum samples were refrigerated until the performance of the ELISA (IDEXX Laboratories, Westbrook, ME, respectively).

In study 1, blood samples were stimulated with specific antigens as described elsewhere (27). All samples were divided into five aliquots and incubated for 18 h with pokeweed mitogen, PBS (blank), PPDa, PPDb and an antigenic cocktail formed by the early secretory antigenic target-6 (ESAT-6) and the culture filtrate protein 10 (CFP-10), two highly specific *M. bovis* antigenic proteins (28). Samples were then centrifuged and the supernatant was analyzed using the Bovigam 2.G (Prionics, Schlieren-Zurich, Switzerland) according to the manufacturer's recommendations. Two criteria based on different sets of antigens were applied to classify animals as positive; for criteria A (IGRAb) animals were considered positive if the optical density (OD) obtained after stimulation with PPDb (OD_PPDb_) minus the OD of the aliquot stimulated with PBS (OD_PBS_) was ≥0.1 and OD_PPDb_-OD_PPDa_ ≥0.1; in the case of criteria B (IGRAc), animals were classified as positive when OD_cocktail_-OD_PBS_ was ≥0.1. For study 2 a commercial ELISA (IDEXX Laboratories, Westbrook, ME) was used to detect MPB83 and MPB70 bTB specific antibodies as described elsewhere (19). Animals with an S/P ratio ≥ 0.3 were considered positive and negative if else as recommended by the manufacturer.

### Statistical Models

Latent-class models were used to estimate diagnostic test accuracy (sensitivity –Se-, and specificity -Sp-) of the IGRA using the different antigens (IGRAb and IGRAc) and the ELISA in the absence of a gold standard assay (1, 29). Samples collected were assumed to originate from a single population given they were drawn from herds belonging to the same company with similar animal health status regarding bTB and similar production management standards.

For each study (1 and 2) two different models were used alternatively: a two dependent tests-one population model (LCA-a) using the results from the skin test (CFT or CFT-CCT) and one of the *in-vitro* tests (IGRA or ELISA), and a one test-one population model (LCA-b) analyzing the results of the *in-vitro* tests separately (Figure 1).

Conditional correlation coefficients for the Se (rhoD) and Sp (rhoDc) were included in the LCA-a models as described elsewhere (29). We assumed results from the tests were conditionally dependent because results from diagnostic tests targeting a similar biological phenomenon, such as the intradermal tests and the IGRA (30), are likely dependent (29, 31). Similarly, and although the ELISA is based on the detection of the humoral immune response in the infected animals, there is a relationship between the initial predominant cellular-mediated immunity and the posterior humoral immunity observed as disease progresses in the animal (32), so results from the skin test and the ELISA were also assumed to be conditionally dependent.

Beta prior distributions for the Se and Sp of the CFT, CFT-CCT, IGRAb, IGRAc, and ELISA were chosen according to previous reports (Table 1, Supplementary Table 1). Distributions were fitted using Beta buster version 1.0 (downloadable at https://cadms.vetmed.ucdavis.edu/diagnostic/software). More informative distributions were used for the Se and Sp of the CFT-CCT due to the availability of Uruguay-specific information (40) compared with those used for the *in-vitro* assays, since most references for those originated from other countries with a different experience in the use of these techniques (Table 1).

**Table 1:** Prior estimates (Mode and 5th percentiles) for sensitivity, specificity of the intradermal tests (CFT, CFT-CCT) and *in-vitro* (IGRAb, IGRAc, and ELISA) bTB tests, and prevalence for the two models implemented.

**Diagnostic test**	**Priors estimates**				**References**
	**Sensitivity**	**Beta distribution**	**Specificity**	**Beta distribution**	
CFT	80 (>51)	α: 7.99, β: 2.75	90 (>60)	α: 8.3045, β: 1.81	(33–39)
CFT-CCT	53 (>46)	α: 73.81, β: 65.57	97 (>94)	α: 176.39, β: 6.42	(33, 36, 40, 41)
IGRAb	83.5 (>48)	α: 5.99, β: 1.99	95 (>80)	α: 21.20, β:2.06	(5, 18, 32, 42–44)
IGRAc	80 (>60)	α: 14.84, β: 4.46	97 (>94)	α: 176.39, β: 6.42	(36, 42, 45)
ELISA	57.1(>33.1)	α: 6.98, β: 5.49	95 (>81)	α: 23.25, β: 2.17	(4, 19, 46, 47)
Prevalence(^*^)	35 (>15)	α: 3.63, β: 5.88		Experts opinion	
Prevalence (+)	85 (>61)	α: 8.46, β: 1.742		CFT-estimated	

(*) * Prevalence priors distributions based on expert opinions used in the LCA-a*.

*(+) Prevalence priors distributions based on results from the intradermal test (CFT) used in the LCA-b*.

For the LCA-a (two-dependent-test) models, prevalence priors were formulated from expert opinion following procedures described elsewhere (3). For the LCA-b (one-test) models, prior distributions for prevalence were formulated using the results from the CFT-CCT as described previously (48). Briefly, we simulated the true prevalence distribution using the Rogan-Gladen estimation method to correct for the imperfect Se and Sp of the CFT-CCT (assumed to follow beta distributions as mentioned before) (Table 1) through 5,000 iterations in an Excel spreadsheet (Microsoft Office Professional Edition, 2016) using @Risk software version 7.0.0 (Palisade Corporation 2015). The outputs from the simulations were used to fit a beta distribution that was used as the prevalence prior for LCA-b models.

Three Markov chain Monte Carlo runs were implemented per model to visually assess convergence (also tested using the Gelman-Rubin^∧^R statistic) (49). Models were run for 7,500 iterations for computing posterior estimates after an initial burn-in of 2,500 samples. To eliminate potential autocorrelation we applied thinning and selected one every 10 consecutive samples. Latent-class models were fitted using OpenBUGS 3.2.2 (50) via the R2OpenBUGS package (51) from the R 3.2.4 software. The influence of the selected priors on the posteriors distributions was evaluated by comparing the initial models with a model fitted using non-informative uniform (0,1) distributions for each parameter under evaluation. The possible independence between the results of the two tests being assessed was also evaluated by fitting models that did not include correlation terms. Model fit was assessed using the deviance information criterion (DIC), and the model selection (LCA-a or LCA-b) was based on lower DIC (52) and narrower posterior credibility intervals.

## Results

Cross-tabulated dichotomous results for the combination of the intradermal tests (CFT or CFT-CCT), and the *in-vitro* assays (IGRAb, IGRAc, or ELISA) are presented in Table 2. Animals with missing IGRAs diagnostic results followed similar proportion of CFT results than those used for the analyses (Pearson's chi-square 2.91, *P* > 0.05).

**Table 2:** Cross-tabulated dichotomous diagnostic results for intradermal test (CFT, CFT-CCT) and *in-vitro* (IGRAb, IGRAc, ELISA) bTB- diagnostic tests.

**Study**	**Diagnostic test**		**CFT+**	**CFT-**	**CFT-CCT+**	**CFT-CCT-**	**Total**
1	IGRAb	Positive	35	19	26	28	54
		Negative	25	42	10	57	67
	IGRAc	Positive	34	16	24	26	50
		Negative	26	45	12	59	71
		Total	60	61	36	85	121
2	ELISA	Positive	126	3	91	38	129
		Negative	108	42	64	86	150
		Total	234	45	155	124	279

The estimated posterior estimates for the Se and Sp of the diagnostic tests and the prevalence in Study 1 and 2 are shown in Table 3.

**Table 3:** Posterior estimates (median and 95% posterior probability interval) for CFT, CFT-CCT and *in-vitro* assays (IGRAb, IGRAc, ELISA) sensitivities, specificities, prevalence, and, when applicable, correlation terms (rhoD, rhoDc) distributions obtained for study 1 (121 animals) and study 2 (279 animals), applying the model “a,” or the model “b” in chronic naturally infected dairy herds in Uruguay.

**Study/Model**	**Diagnostic test**			**Posteriors estimates**				
	**Test-one**	**Test-two**	**DIC**	**Sensitivity**	**Specificity**	**Prevalence**	**rhoD**	**rhoDc**
**1/a**		IGRAb		75.32 (58.96, 91.63)	89.96 (77.82, 97.23)^*^	50.84 (33.80, 67.73)	−4.09 (−28.94, 35.07)	−2.78 (−20.70, 23.68)
	CFT		19.4	73.34 (56.88,89.44)	77.02 (58.96, 95.48)			
		IGRAc	19.4	75.73 (62.45, 88.08)	96.49 (93.85, 98.22)^*^	51.33 (38.11, 65.33)	−3.50 (−24.00, 24.73)	−0.33 (−7.84, 9.23)
	CFT			72.43 (58.34, 83.75)	76.23 (59.98, 93.95)			
**2/a**		ELISA	19.4	57.82 (48.92, 73.43)	93.76 (85.57, 98.08)	76.94 (56.97, 87.80)^*^	11.17 (−1.96, 29.72)	4.75 (−1.39, 27.78)
	CFT			95.48 (88.83, 98.91)	63.87 (34.15, 94.31)			
**1/a**		IGRAb	18.1	78.01 (62.97, 89.53)	91.43 (78.91, 98.26)	50.37 (37.38, 63.48)	−2.47 (−31.69, 29.59)	−0.48 (−8.19, 16.20)
	CFT-CCT			53.27 (45.76, 60.59)	96.19 (92.78, 98.37)			
		IGRAc	17.7	76.21 (65.35, 85.86)	96.56 (93.34, 98.52)	51.30 (40.28, 62.97)	−6.03 (−28.49, 17.95)	−0.09 (−3.42, 5.10)
	CFT-CCT			52.89 (45.66, 59.93)	96.13 (92.66, 98.32)			
**2/a**		ELISA	24.4	52.29 (44.96, 60.35)	92.41 (78.82, 98.48)^*^	79.73 (73.23, 91.80)^*^	17.05 (−0.26, 31.64)	−0.08 (−8.02, 16.78)
	CFT-CCT			60.44 (54.45, 66.59)	96.14 (92.60, 98.34)^*^			
**1/b**		IGRAb	7.3	58.12 (43.14, 86.23)	92.70 (77.84, 98.85)^*^	76.57 (48.06, 96.68)	NA	NA
		IGRAc	7.8	66.04 (46.97, 86.68)	96.72 (93.54, 98.68)^*^	65.37 (45.68, 91.79)	NA	NA
**2/b**		ELISA	8.4	53.85 (44.41, 71.28)	92.42 (78.63, 98.76)^*^	83.79 (59.92, 97.78)	NA	NA

*Model “a”: Two-dependent-test and one population model*.

*Model “b”: One-test one population model*.

*IGRAb: Interferon gamma release assay using PPDb-PPDa antigens*.

*IGRAc: Interferon gamma release assay using peptide cocktail antigens*.

*ELISA: Commercial Enzyme-immunosorbent assay*.

* *Differences between the use of informative vs. uniform priors reflects a >10.5% variation in the posterior estimates*.

### Study 1

Median posterior estimates for the prevalence, Se and Sp of the intradermal tests (CFT, and CFT-CCT) using the LCA-a model were similar regardless the antigen used in the IGRA (IGRAb or IGRAc) (Table 3). The median posterior IGRAb Sp estimates were slightly lower than those obtained for the IGRAc, whit higher median Sp values for the models integrating CFT-CCT as second test as well, but with the overlapping of the PPIs (Table 3).

LCA-b models consistently yielded lower Se values for both IGRAs and higher prevalence estimates compared with LCA-a models, but with similar Sp posterior estimates.

### Study 2

The LCA-a model yielded higher posterior estimates for the prevalence and Se of the intradermal tests, and a markedly lower Sp posterior values for CFT compared to those observed in study 1 using the same model. ELISA Se and Sp estimates obtained using the two models (LCA-a and b) were consistent.

Conditional correlation between intradermal and *in-vitro* test results in infected (rhoD) and non-infected animals (rhoDc) was low, with a 95% Posterior Probability Interval (95%PPI) including 0 in all LCA-models for study 1 (Table 3). However, no significant improvement was observed in the DIC when test independence was assumed for models using IGRAs and CFT (study 1: 19.4 vs. 19.4, 19.4 vs. 19) or IGRAs and CFT-CCT (study 1: 18.1 vs. 17.5, 17.7 vs. 19.7) respectively. Interestingly, the LCA-a model from study 2 showed the highest median correlation terms for infected animals (rhoD = 11.7 and 17.05), showing a poorer fit of the model when independent-tests models were assessed (ELISA and CFT DIC:19.4 vs. 24.7, ELISA and CFT-CCT DIC:24.4 vs. 31.9), although 95% PPI included 0.

The sensitivity analysis revealed that results obtained using LCA-a models for study 1 were not affected (changes <10.5%) by the use of weakly informative priors (Supplementary Table 2). However, various parameters were severely affected (changes>10.5% when weakly informative priors were used) by the choice of priors in the remaining models/studies. Results were most affected when LCA-a models were applied in study 2. The use of uniform distributions for the Sp of the *in-vitro* assays in both studies resulted in 17.8 to 38.2% decreased posterior median Sp values. Similarly, use of uniform priors for the prevalence resulted in a >20% reduction in posterior estimates of study 1 using the LCA-b model (76.5 to 54.3 and 65.4 to 52.2), and an increase in the Se estimates for IGRAb and IGRAc.

All models reached convergence as indicated by the visual inspection of the Markov chains and the Gelman-Rubin∧R statistic (<1.002) for all parameters.

## Discussion

Due to the increasing number of bTB- infected herds in Uruguay (Animal Health Bureau, Uruguay -DSA MGAP-), the need for early and accurate detection, isolation and removal of infected animals from a herd is crucial when whole herd-culling is not an economically or socially sustainable option. Here, we aimed to assess the performance of bTB-*in-vitro* assays under field conditions for the first time in Uruguay with the ultimate goal of improving current bTB diagnostic strategies for chronic and high prevalence infected dairy herds.

In order to estimate the performance of the *in-vitro* assays evaluated here we used LCA, a suitable analytical approach when no reliable gold standard is available (1, 53, 54), as it is the case for bTB (5, 6, 55). We fitted two different latent-class models using prevalence priors based on expert opinion or diagnostic test results in order to evaluate the potential impact of a given methodological approach. Based on DIC models with three (Se, Sp, Prev) parameters were preferred above those with seven (Se_1_, Se_2_, rhoD, Sp_1_, Sp_2_, rhoDc, Prev). Correlation between test results were very low in all models/test pairs, what had been already described for the IGRA and single skin test (5, 6) such result is expected because the diagnostic tests evaluated here have high Sp (1). However, the comparatively higher correlation between the ELISA and CFT or CFT-CCT estimates in bTB-infected animals (rohD) was surprising, given that the ELISA and the skin tests target different immune responses and therefore a larger degree of independence is often assumed (32, 56).

Prevalence priors elicited from expert opinion were considerably lower than those based on the intradermal test results (median of 0.35 vs. 0.85). That finding could explain, at least in part, the lower posterior estimates for prevalence obtained in LCA-a models compared with those from LCA-b models. The higher posterior prevalence estimates obtained using all models in both studies; along with the fact that the two sampled herds remained infected with high rates of reactors 2 years after this study was completed (data not shown) suggest that bTB-infection was higher than what was estimated using expert opinion in this population. Comparison of results from the two modeling approaches illustrates the potential negative consequences of basing prior distributions exclusively on expert opinion.

Interestingly, estimates for the Se and Sp of the CFT test were lower than those described for the US (33, 34, 37), and more in line with Se values reported in field studies in Australia (35). Likewise, posterior estimates for the serial use of CFT-CCT, remained in the lower end of previous estimates (33, 36, 40, 41). This relatively low accuracy of the intradermal tests in Uruguay suggests that the bTB-control program may suffer from limited Se in heavily infected herds (~53%), what could lead to the persistence of infected animals in the dairy cattle population over time. Possible explanations for this finding include: presence of other infections that could compromise the reliability of bTB diagnostic tests such as Johne's disease, that is prevalent in dairy herds in Uruguay and whose impact on bTB diagnosis has been already suggested in the country (14), a high proportion of animals in an advanced stage of disease (state of anergy) (30, 57), what could be plausible given the high prevalence of infection in the tested herds, along with other factors associated with the performance of the technique itself or the animals tested, which, with the consolidation and intensification of the industry, may have contributed to the re-emergence of bTB observed in the last decade (14).

Posterior estimates for the Se of IGRAb and IGRAc obtained using LCA-a models (Table 3) are in agreement with previous reports suggesting IGRAs are at least as sensitive as intradermal assays (32, 58). IGRAs have two major advantages over intradermal tests, namely, the potential for detecting false negative animals in the skin test (41, 59, 59–61), and the opportunity to maximize their sensitivity thanks to the anamnestic effect induced by the inoculation of PPDs when used in combination with intradermal tests (18, 41, 62). The population under study was sampled a post intradermal inoculation of the PPDb, while this time was variable, it could have contributed to an enhanced Se in agreement with previous studies in which IGRAs performance was assessed following intradermal tuberculin testing (63).

The low Se estimated for the intradermal testing protocol currently used in the bTB-control program in Uruguay (i.e., CFT-CCT) suggests that *in-vitro* tests might be advantageous if used in parallel to improve the Se of the program in these heavily infected herds. Assuming independence among diagnostic tests, as indicated by the correlation coefficients (rhoD and rhoDc) including zero in the dependent models, an estimate of the potential overall Se of the combination in parallel can be computed using the median estimates values as 1–[1–Se(*in-vivo*)] ^*^ [1-Se(*in-vitro*)] (64). This approximation showed a 19–25% improved Se in the different *in-vivo* and *in-vitro* combinations (Supplementary Table 3) which could vastly improve the detection of bTB-infected animals. A slightly higher Sp was obtained for the IGRAc compared with the IGRAb, what could be due to the use of more specific antigens (peptide-cocktail with ESAT-6 and CFP-10) (28, 42, 45, 46) although could be also a product of the different priors used for each test based on available knowledge. Interestingly, Sp of the IGRAc was equivalent to that of CFT-CCT, suggesting that the use of a single assay (IGRAc) could potentially replace serial testing with CFT-CCT for bTB screening in heavily infected dairy herds without increasing the rate of false positives compared with the current strategy in the project, thus avoiding the unnecessary culling of non-infected cattle.

Care should be taken when interpreting our results, since these were obtained in herds with a very high bTB prevalence and therefore may not be easily extrapolated to other epidemiological situations in Uruguay. The low sensitivity of the skin tests found here could be related with the presence of a larger proportion of infected animals in an advanced stage that is expected in herds with a high infectious pressure, as the ones evaluated here. Therefore, the gain in sensitivity that could be expected from the ancillary application of *in-vitro* tests (Supplementary Table 3) may not be as high in other situations (smaller dairy herds with lower infection levels, beef herds, etc.). Furthermore, other herd-level factors (such as the presence of Johne's disease) could have also contributed to the results found here. Still, highly infected large dairy herds are one of the main issues faced by the bTB eradication program and therefore our priority here was to assess the usefulness of additional diagnostic approaches to complement the current diagnostic strategy. Additional measures to control the spread of bTB in the Uruguayan cattle population include ban of animal movement, more frequent bTB-testing, the pasteurization of milk use for pre-weaned calves.

In conclusion, results found here, irrespective of the modeling approach followed, suggest that the use of IGRAs in Uruguay can dramatically improve the limited Se of the currently used diagnostic strategies based on skin tests, which would require numerous herd tests to eliminate disease from heavily infected dairy herds as the ones analyzed here. The ELISA could also have some potential for detection of bTB-infected animals if used as an ancillary test to skin test in these populations.

## Data Availability

All datasets generated for this study are included in the manuscript and/or the Supplementary Files.

## Ethics Statement

This study was performed in accordance with guidelines of the animal ethics committee of the University of Uruguay (Comision Honoraria de Experimentacion Animal).

## Author Contributions

CP-R, JA, and AP designed the study and drafted the manuscript. AS, XS, and AN coordinated sample collection, field testing, and laboratory analysis. AG assembled the databases and helped in interpretation of the results. CP-R run the statistical analyses. All authors critically revised the manuscript.

### Conflict of Interest Statement

The authors declare that the research was conducted in the absence of any commercial or financial relationships that could be construed as a potential conflict of interest.
