# Supplementary material for: Modeling the Accuracy of Two in-vitro Bovine Tuberculosis Tests Using a Bayesian Approach
Source: Front Vet Sci. 2019 Aug 13;6:261. doi: 10.3389/fvets.2019.00261 (PMC6701407; doi:10.3389/fvets.2019.00261)
Supplement: Supplementary file 1 [file Table_1.pdf]

Supplementary table 1. References for the elaboration of prior distributions for sensitivity (Se) and specificity (Sp) for intradermal (CFT, CFT-CCT) and in-vitro (IGRAb, IGRAc, ELISA) diagnostic tests evaluated**.**

Test	Antigen	Cutoff	Se	95CI		Sp	95CI		Characteristics	Reference (et al.)	Year	Origin
CFT		Any	76	56	89	100	92	100	Meta-analisis	Nuñez-Garcia	2018	UK & Ireland
		palpable	85.7			92.6			Meta-analisis	Farnaham	2011	USA
		increase	83.33	51.59	97.91				Study	Norby	2004	USA
			80.4						Study	Whipple	1995	USA
			82			96			Review	USDA-APHIS	1992	USA
			65.6	56.6	73.9				Study	Wood	1991	Australia
			81.8			96.3			Study	Francis	1978	Australia
CFT-CCT		>4mm	50	26	78	100	99	100	Meta-analisis	Nuñez-Garcia	2018	UK & Ireland
			53	46	62				Study	VanderWaal	2017	Uruguay
			55.1-93.5			88.8-100			Review	Vordemeier	2006	Global
			75	42.81	94.51				Study	Norby	2004	USA
IGRA	PPDb-	0.1	60.7	48	72				Study	Casal	2014	Spain
	PPDa		83.5	73.6	91.6	90.4	89.1	92.7	Study	Alvarez	2012	Spain
	(IGRAb)		87.6	73	100	96.6	85	99.6	Review	de la Rua Domenech	2006	Global
			88			95			Study	Gomley	2006	Ireland
			66			84			Study	Aagaard	2006	Global
			85	72	90	93	89	96	Study	Ryan	2000	New Zealand
IGRA	Peptide-	0.1	78	60	90	99	99	100	Meta-analisis	Nuñez-Garcia	2018	UK & Ireland
	Cocktail		80			100			Study	Flores-Villalba	2012	Mexico
	(IGRAc)		85	73	94	97	94	100	Study	Aagaard	2006	Global
ELISA	MPB83-	>0.3	61.1	33.1	84.6	85.4	81.7	88.8	Study	Al-Mouqatea	2018	Kuwait
	MPB70		57.1	44	69	100			Study	Casal	2012	Spain
			18.1			96.4			Study	Wood	1992	Australia
			61.9	30	96.7	98.2	93.8	100	Review	Waters	2012	UK,Ireland,NZ,USA

Supplementary table 2. Results from the sensitivity analyses using uniform distributed priors for sensitivity (Se), specificity (Sp), and Prevalence (prev) for each bTB-diagnostic test evaluated.

Reference values indicate posterior median results and posterior probability intervals (LowPPI, HighPPI) for the model including informative priors.

The table is shown in the Excel file.
