# Supplementary material for: Modeling the Accuracy of Two in-vitro Bovine Tuberculosis Tests Using a Bayesian Approach
Source: Front Vet Sci. 2019 Aug 13;6:261. doi: 10.3389/fvets.2019.00261 (PMC6701407; doi:10.3389/fvets.2019.00261)
Supplement: Supplementary file 3 [file Table_3.pdf]

Supplementary table 3. Median posterior sensitivity and specificity estimates for parallel test combination between in-vivo (CFT or CFT-CCT) and in-vitro (IGRAb, IGRAc, ELISA) bTB diagnostic tests.

**Test-one in-vivo**	**Test-two in-vitro**	**Posteriors median estimates**		**Parallel estimates**	
		Sensitivity	Specificity	Sensitivity	Specificity
CFT		0.733	0.770		
	IGRAb	0.753	0.900	0.934	0.693
	IGRAc	0.757	0.965	0.935	0.736
	ELISA	0.578	0.938	0.888	0.599
CFT-CCT		0.533	0.962		
	IGRAb	0.780	0.914	0.897	0.879
	IGRAc	0.762	0.966	0.889	0.928
	ELISA	0.523	0.924	0.777	0.888

CFT: Caudal Fold Tuberculin Test
CFT-CCT: CFT and Comparative Cervical Tuberculin Test combined in series
IGRAb: Interferon-gamma release assay using PPDb-PPDa antigens
IGRAc: Interferon-gamma release assay using peptide cocktail antigens
ELISA: Commercial Enzyme-immunosorbent assay
